# Circulating Sclerostin and Irisin Are Related and Interact with Gender to Influence Adiposity in Adults with Prediabetes

**DOI:** 10.1155/2014/261545

**Published:** 2014-09-03

**Authors:** Theerawut Klangjareonchai, Hataikarn Nimitphong, Sunee Saetung, Nuttapimon Bhirommuang, Rattanapan Samittarucksa, Suwannee Chanprasertyothin, Rattana Sudatip, Boonsong Ongphiphadhanakul

**Affiliations:** ^1^Department of Medicine and Research Center, Ramathibodi Hospital, Mahidol University, Rama VI Street, Rajathevi, Bangkok 10400, Thailand; ^2^Division of Endocrinology and Metabolism, Department of Medicine, Faculty of Medicine, Ramathibodi Hospital, Mahidol University, Rama 6 Road, Rajathevi, Bangkok 10400, Thailand

## Abstract

*Objectives.* Sclerostin, an osteocyte-specific protein, has been found to be related to adiposity and glucose metabolism. Irisin, a myokine, can affect browning of white fat and influence glucose and energy homeostasis. Taken together, this suggests a probable network among fat, bone, and muscle that may influence health outcomes. The aims of this study were to investigate the relationship of circulating sclerostin and irisin and their association with adiposity (assessed by body mass index (BMI)). *Materials/Methods.* A cross-sectional study included 98 adults with impaired fasting glucose and/or impaired glucose tolerance. 75 gm OGTT was performed in all subjects. Fasting plasma samples were obtained for glycated hemoglobin, calcium, creatinine, serum sclerostin and irisin. *Results.* Circulating irisin and sclerostin were highly correlated (*r* = −0.4; *P* < 0.001). After controlling for age, gender, and BMI, irisin was significantly related to sclerostin (*P* < 0.001). Multivariate linear regression analysis demonstrated that circulating sclerostin (*β* = −0.45; *P* < 0.05) and irisin (*β* = −0.46; *P* < 0.05) were negatively associated with BMI, independent of age in males. In females, no relationship of sclerostin or irisin to BMI was found. *Conclusions.* Circulating irisin and sclerostin are highly related. Interventions targeting irisin could affect sclerostin and vice versa.

## 1. Introduction

Multipotential mesenchymal stem cells (MSCs) are able to differentiate into adipocytes, myocytes, and osteoblasts. Canonical Wnt or Wnt/*β*-catenin signaling regulates MSC differentiation [1]. Activation of canonical Wnt signaling promotes differentiation of MSCs into myoblasts and osteoblasts but inhibits differentiation into preadipocytes [2]. Canonical Wnt signaling organizes the balance among adipogenesis, myogenesis, and osteogenesis [2].

Sclerostin is produced by osteocytes and inhibits the Wnt signaling pathway, which in turn leads to the inhibition of osteoblast differentiation and bone formation [3]. Moreover, Wnt signaling blocks adipogenesis through suppression of CCAAT/enhancer-binding protein-α (CEBPα) and peroxisome proliferator-activated receptor-*γ* (PPAR*γ*) and blocks the thermogenic program through suppression of PPAR*γ* coactivator-1α (PGC-1α) [2]. Consequently, sclerostin stimulates adipogenesis or fat production. In humans, a positive correlation has been reported between circulating sclerostin and the percentages of abdominal, gynoid fat [4], and fat mass [5]. In addition, type 2 diabetic subjects had higher circulating sclerostin than in nondiabetic control subjects [6, 7]. Sclerostin is also associated with the duration of type 2 diabetes mellitus (T2DM) and the level of glycated hemoglobin (HbA1c) in T2DM patients [8]. To date, there have been reports of positive correlations between sclerostin and male gender, age, body mass index (BMI), and T2DM and negative correlations between sclerostin and estrogen replacement, mechanical unloading, and intermittent parathyroid hormone (PTH) therapy [4, 8–12].

Irisin is a recently identified myokine released into the blood circulation during exercise [13]. Irisin is secreted in response to PGC-1α activation; this hormone induces the transformation of white fat cells into beige fat cells. This type of fat functions, as brown fat, increases total body energy expenditure, reduces body weight, and increases insulin sensitivity [13, 14]. In humans, existing data of irisin levels in relation to obesity, diabetes, and metabolic syndrome are inconclusive [15]. For examples, an epidemiologic study demonstrated that circulating irisin levels were lower in nondiabetic, overweight, and obese subjects compared to lean subjects [16]. In addition, with comparable BMI, subjects with T2DM had lower levels of circulating irisin than controls [16]. On the contrary, there was a trend of positive association between circulating irisin and BMI in a study of healthy women [17, 18]. Nonetheless, this association disappeared after adjusting for potential confounders [17]. Other existing data have demonstrated positive correlations between irisin and estradiol level, muscle mass, and insulin sensitivity [16, 17]. On the other hand, older age and fat mass were negatively correlated with irisin [16, 17]. Taken together, this suggests a crosstalk among bone and fat though sclerostin and among muscle and fat through irisin. However, no data exists regarding a direct relationship between sclerostin and irisin. Therefore, the aim of the present study was to investigate the relationship of circulating irisin and sclerostin and their association with adiposity as assessed by BMI in adults at high risk of T2DM.

## 2. Materials and Methods

### 2.1. Study Population

This cross-sectional study was conducted at Ramathibodi Hospital, Mahidol University, Bangkok, Thailand. Two hundred and forty healthy volunteers who aged 35–80 years were recruited by advertisement for the screening of type 2 diabetes from July 2012 to March 2013. A 75 g oral glucose tolerance test (OGTT) was performed in the morning after an 8 h overnight fast to recruit subjects with impaired fasting glucose (IFG) and/or impaired glucose tolerance (IGT) according to American Diabetes Association criteria [19]. There were 98 subjects with IFG and/or IGT included in this study. Other inclusion criteria were adults with normal renal function, hepatic function, and calcium level. Exclusion criteria were known diseases affecting bone (hyperparathyroidism, hypercortisolism, renal bone disease, and chronic liver disease) and previous or current treatment with drugs affecting bone metabolism (calcium supplements, vitamin D supplements, selective estrogen receptor modulators, estrogen therapy, calcitonin, antiresorptive therapy, thiazides, glucocorticoids, and anticonvulsants). The ethical review board of Ramathibodi Hospital, Mahidol University, approved this study; all participants provided written informed consent.

### 2.2. Clinical Evaluation

The height of each subject was measured to the nearest 0.1 cm using a stadiometer. Body weight was measured using a digital weighing scale (Soehnle 7755; Nassau, Germany). Body mass index (BMI) was calculated by the Quetelet formula: weight in kilograms divided by the square of height in meters (kg/m^2^). Waist circumference (WC) was measured at the umbilical level.

### 2.3. Laboratory Assays

Samples of venous blood were taken in the morning after fasting overnight. Serum was stored at −80°C until examination. Fasting plasma glucose (FPG), 2 h plasma glucose (2 hPG), glycated hemoglobin (HbA1c), calcium and creatinine were measured using standard automated laboratory techniques.

Serum sclerostin was measured using a quantitative ELISA developed by Biomedica (Vienna, Austria). The intra-assay variability was 5% and the interassay variability was 3%. Serum irisin was measured using a quantitative ELISA developed by AdipoGen (Incheon, South Korea). The expected range in human serum according to the manufacture is 0.2–2 *μ*g/mL. There is no report of crossing reactivity with molecule other than irisin. The intra-assay variability was 6.7%, and the interassay variability was 8%.

### 2.4. Statistical Analysis

Variables were presented as mean ± standard error of mean (SEM), unless stated otherwise. The Kolmogorov-Smirnov test was used to assess the normality of distribution of continuous variables. All parameters were in normal distribution. Differences between males and females were determined by unpaired Student's *t*-test. Pearson's correlation coefficients described associations between continuous variables. Multivariate linear regression analyses of covariance were used to evaluate the influence of independent variables on serum sclerostin levels or BMI. A *P* value < 0.05 was considered statistically significant.

## 3. Results

### 3.1. Baseline Characteristics of the Study Population

Table 1 shows the clinical characteristics and biochemical parameters of the study population. There were 25 males and 73 females with a mean age of 60 years. When comparing males and females, there were no differences in age, BMI, WC, FPG, 2 hPG, and HbA1c. Circulating irisin was found to be higher in females than in males (3.08 ± 0.07 versus 2.67 ± 0.12 *μ*g/mL; *t* = 2.9; df = 96; *P* < 0.01). On the other hand, circulating sclerostin tended to be higher in males (83.38 ± 5.59 versus 70.52 ± 3.49 pmol/L; *t* = 1.9; df = 96; *P* = 0.06).

### 3.2. Relationship between Sclerostin and Irisin Levels

**Figure 1:**
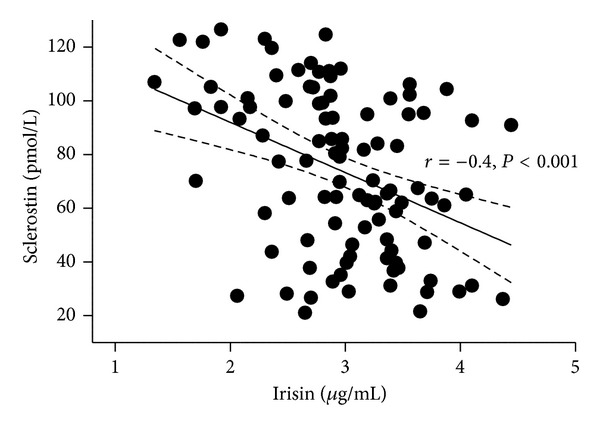
Relationship between circulating sclerostin and irisin in the study population (*n* = 98).

Circulating sclerostin and irisin were highly correlated (*r* = −0.4; *P* < 0.001) (Figure 1).  Table 2 shows the correlation matrices between sclerostin, irisin, and other relevant variables in males and females. Sclerostin and irisin are negatively correlated in both males (Table 2(a)) and females (Table 2(b)). Sclerostin increased with advancing age only in females (Table 2(b)). After controlling for age, gender, and BMI, irisin was still significantly related to sclerostin, as shown in Table 3.

### 3.3. Multivariate Models including BMI and Serum Sclerostin, Irisin and Age

Multivariate linear regression analysis demonstrated that circulating sclerostin (*β* = −0.45; *P* < 0.05) and irisin (*β* = −0.46, *P* < 0.05) were negatively associated with BMI, independent of age in males (Table 4(a)). In females, no relationship of sclerostin or irisin to BMI was found (Table 4(b)).

## 4. Discussion

The conventional view of the muscle-bone relationship is that skeletal muscle exerts physical loading on the skeleton and that bone provides an attachment site for skeletal muscle. Beyond that, the knowledge regarding a likely biochemical crosstalk between skeletal muscle and bone is limited [20]. Recently it has been reported that beige fat is anabolic to bone [21]. A study of transgenic mice with the transcription factor forkhead box C2 (FoxC2) that promotes brown fat development indicated that inducible beige fat downregulates the expression of sclerostin. It is likely that the negative association between irisin and sclerostin may be mediated through the influence of irisin on the stimulation of the browning of adipocytes [21]. To our knowledge, the present study is the first report that reveals a correlation of serum sclerostin level with serum irisin. Sclerostin plays a role in bone and fat cell formation [2, 3], while irisin plays a well-established role in beige fat cell formation [13]. Taken together, this suggests an interrelationship among fat, bone, and muscle. It is of note that bone, adipose tissue, and skeletal muscle are tissues responsible for high-burden diseases associated with aging, such as osteoporosis, obesity, and sarcopenia. These disorders often occur concurrently and contribute to increases in morbidity and mortality as well as impaired quality of life in the elderly. Together with the results in the present study, it appears that these tissues can often affect one another directly or indirectly through their secretory products. Because of these interacting links of their secretory products among bone, adipose tissue, and skeletal muscle, it complicates the effort to identify the secretory products which are directly causal from clinical study data. Identifying links among secretory products and discriminating those that are direct will enhance our understanding of the interactions and will likely bring about therapeutic measures with fewer adverse effects on other tissues and with simultaneous benefits for more than a single disease. Further more robust studies are needed to systematically identify these interacting links and their causal relationships.

We also demonstrated in this study that age, irisin, and sclerostin were independently associated with adiposity (as assessed by BMI) only in males. Both irisin and sclerostin have been separately reported to be associated with adiposity [4, 16]. Previous studies have shown that irisin mRNA is decreased in both adipose tissue and muscle in obese subjects [16]. There are conflicting data about the relationship between irisin and adiposity as there were reports of both negative [16] and positive correlations [17, 18]. In our study, the negative correlation between irisin and BMI when controlling for age and irisin in males subjects is similar to the findings in the study of Moreno-Navarrete et al. [16]. Additional studies with the larger sample size are needed to elucidate the physiological role of irisin in obesity. On the other hand, sclerostin was found to be positively related to BMI or other measures of adiposity in studies of nondiabetic subjects [4, 5, 22]. However, no significant relationship between sclerostin and BMI was detected in a study of subjects with type 2 diabetes [8]. Likewise, no relationship between sclerostin and BMI was found in our prediabetes study population. It thus appears that glucose tolerance status may influence the relationship between sclerostin and adiposity. However, when controlling for irisin, there was a significant relationship between sclerostin and BMI in males, but the direction of association was opposite to that reported in nondiabetic subjects. This may be suggestive of the existence of the influence of irisin on BMI, or vice versa, in subjects with prediabetes, which should be taken into account when trying to determine the unconfounded relationship between sclerostin and adiposity in abnormal glucose tolerance status.

In the present study, we found a sexual dimorphism in terms of BMI in relation to serum irisin and sclerostin of which the underlying basis is not entirely clear. However, it is well established that sexual dimorphism exists for body composition. In general, adult males have higher lean mass and bone mass, but a lower fat mass than females after corrected for body size [23]. Such difference is likely to be attributable to sex steroid hormones and probably their interaction [24]. Moreover, estradiol level may influence serum sclerostin and irisin [17, 25]. Based on the complex interrelationship among sex steroid hormones, body composition, and irisin, as well as sclerostin, the relationship between BMI and sclerostin as well as irisin could be different according to gender, as shown in our study.

Our data confirmed previously have shown positive relationships between age and sclerostin levels in both genders. Previous studies reported that at any given total body bone mineral content and presumably an equivalent number of osteocytes, serum sclerostin levels were higher in the elderly compared to young subjects. This is suggesting that with aging, there is an increased sclerostin production by individual osteocytes. Nonetheless, the reduction in sclerostin clearance with aging could not be entirely excluded [8, 12]. Moreover, our study also confirmed previous findings that males have higher serum sclerostin levels and lower serum irisin levels than females; this is in accordance with recent studies showing that sclerostin and irisin are partly regulated by estrogen [17, 25–27].

The present study has a number of limitations. Our study shows only association. The causal relationship between sclerostin and irisin still remains to be determined. The sample is relatively small and may affect the statistical power of our study. It could be a concern that circulating sclerostin may not reflect sclerostin levels in bone. However, bone marrow plasma and peripheral serum sclerostin concentrations are strongly correlated [28]. This suggests that peripheral serum sclerostin may be a good surrogate of sclerostin secreted from osteocytes. Finally, we did not have data about the physical activity indexes of the subjects enrolled. This may be a weakness of the study since both sclerostin and irisin are influenced by physical activity.

In conclusion, this study demonstrates that serum sclerostin is highly correlated with serum irisin in adults with prediabetes, independent of age, gender, and BMI. Also, circulating sclerostin and irisin are negatively associated with BMI, independent of age in males but not females. We postulate that this relationship might be the language of the crosstalk between fat, bone, and muscle. Additional prospective longitudinal studies are warranted to evaluate the effect of serum irisin on serum sclerostin, BMI, and fat mass.

## Figures and Tables

**Table 1:** Clinical characteristics of the study population according to gender.

	Males (*n* = 25)	Females (*n* = 73)	*P* value
Age (years)	61.08 ± 2.49	58.37 ± 1.03	0.32
Body weight (kg)	74.02 ± 2.73	66.24 ± 1.42	0.02
Height (cm)	164.51 ± 1.10	152.42 ± 0.71	<0.001
BMI (kg/m^2^)	27.25 ± 0.83	28.48 ± 0.60	0.22
WC (cm)	95.21 ± 1.99	96.03 ± 1.37	0.74
FPG (mmol/L)	5.83 ± 0.11	5.80 ± 0.06	0.78
2hPG (mmol/L)	8.75 ± 0.16	8.71 ± 0.17	0.90
HbA1c (%)	6.09 ± 0.06	6.04 ± 0.04	0.53
Irisin (*µ*g/mL)	2.67 ± 0.12	3.08 ± 0.07	<0.01
Sclerostin (pmol/L)	83.38 ± 5.59	70.52 ± 3.49	0.06

Data is expressed as mean ± SEM.

BMI: body mass index, WC: waist circumference, FPG: fasting plasma glucose, 2hPG: 2 h plasma glucose, and HbA1c: glycated hemoglobin.

**(a):** 

	Sclerostin	Irisin	Age	BMI	WC
Sclerostin	1				
Irisin	−0.59	1			
	*P* < 0.01				
Age	0.10	−0.17	1		
	*P* = 0.63	*P* = 0.43			
BMI	−0.24	−0.09	−0.55	1	
	*P* = 0.26	*P* = 0.66	*P* < 0.01		
WC	−0.23	−0.08	−0.46	0.90	1
	*P* = 0.27	*P* = 0.70	*P* < 0.05	*P* < 0.001	

BMI: body mass index, WC: waist circumference.

**(b):** 

	Sclerostin	Irisin	Age	BMI	WC
Sclerostin	1				
Irisin	−0.29	1			
	*P* < 0.05				
Age	0.23	−0.17	1		
	*P* < 0.05	*P* = 0.15			
BMI	−0.18	0.07	−0.19	1	
	*P* = 0.13	*P* = 0.55	*P* = 0.10		
WC	−0.05	0.02	−0.08	0.86	1
	*P* = 0.68	*P* = 0.85	*P* = 0.53	*P* < 0.001	

BMI: body mass index, WC: waist circumference.

**Table 3:** Multivariate linear regression model evaluating the association of sclerostin with irisin after controlling for age, gender, and BMI (*n* = 98).

	Standardized coefficient (*β*)	*P* value
Irisin	−0.35	0.001
Age	0.09	0.39
Gender (1 = male, 2 = female)	−0.06	0.52
BMI	−0.15	0.12

BMI: body mass index.

**(a):** 

	Model 1		Model 2		Model 3	
	*β*	*P* value	*β*	*P* value	*β*	*P* value
Age	−0.55	<0.01	−0.53	<0.01	−0.58	<0.01
Sclerostin			−0.18	0.31	−0.45	<0.05
Irisin					−0.46	<0.05

Model 1: independent variable: age.

Model 2: independent variables: age, sclerostin.

Model 3: independent variables: age, sclerostin, and irisin.

**(b):** 

	Model 1		Model 2		Model 3	
	*β*	*P* value	*β*	*P* value	*β*	*P* value
Age	−0.19	0.11	−0.16	0.19	−0.16	0.19
Sclerostin			−0.14	0.24	−0.14	0.26
Irisin					0.001	0.99

Model 1: independent variable: age.

Model 2: independent variables: age, sclerostin.

Model 3: independent variables: age, sclerostin, and irisin.

## Abbreviations

BMI: Body mass index (weight in kilograms divided by the square of height in meters (kg/m^2^))
75 gm OGTT: 75 grams oral glucose tolerance
HbA1c: Glycated hemoglobin
MSCs: Multipotential mesenchymal stem cells
CEBPα: CCAAT/enhancer-binding protein-α
PPAR*γ*: Peroxisome proliferator-activated receptor-*γ*
PGC-1α: PPAR*γ* coactivator-1α
T2DM: Type 2 diabetes mellitus
PTH: Parathyroid hormone
IFG: Impaired fasting glucose
IGT: Impaired glucose tolerance
WC: Waist circumference
FPG: Fasting plasma glucose
2 hPG: 2 h plasma glucose
ELISA: Enzyme-linked immunosorbent assay
SEM: Standard error of mean
FoxC2: Transcription factor forkhead box C2.

## References

[1] Logan CY, Nusse R (2004). The Wnt signaling pathway in development and disease. *Annual Review of Cell and Developmental Biology*.

[2] Christodoulides C, Lagathu C, Sethi JK, Vidal-Puig A (2009). Adipogenesis and WNT signalling. *Trends in Endocrinology and Metabolism*.

[3] Moester MJ, Papapoulos SE, Löwik CW, van Bezooijen RL (2010). Sclerostin: current knowledge and future perspectives. *Calcified Tissue International*.

[4] Urano T, Shiraki M, Ouchi Y, Inoue S (2012). Association of circulating sclerostin levels with fat mass and metabolic disease—related markers in Japanese postmenopausal women. *Journal of Clinical Endocrinology and Metabolism*.

[5] Sheng Z, Tong D, Ou Y (2012). Serum sclerostin levels were positively correlated with fat mass and bone mineral density in Central South Chinese postmenopausal women. *Clinical Endocrinology*.

[6] Gennari L, Merlotti D, Valenti R (2012). Circulating Sclerostin levels and bone turnover in type 1 and type 2 diabetes. *Journal of Clinical Endocrinology and Metabolism*.

[7] Ardawi M-SM, Akhbar DH, AlShaikh A (2013). Increased serum sclerostin and decreased serum IGF-1 are associated with vertebral fractures among postmenopausal women with type-2 diabetes. *Bone*.

[8] García-Martín A, Rozas-Moreno P, Reyes-García R (2012). Circulating levels of sclerostin are increased in patients with type 2 diabetes mellitus. *The Journal of Clinical Endocrinology & Metabolism*.

[9] van Lierop AH, Witteveen JE, Hamdy NAT, Papapoulos SE (2010). Patients with primary hyperparathyroidism have lower circulating sclerostin levels than euparathyroid controls. *European Journal of Endocrinology*.

[10] Gaudio A, Pennisi P, Bratengeier C (2010). Increased sclerostin serum levels associated with bone formation and resorption markers in patients with immobilization-induced bone loss. *Journal of Clinical Endocrinology and Metabolism*.

[11] Ardawi M-SM, Al-Kadi HA, Rouzi AA, Qari MH (2011). Determinants of serum sclerostin in healthy pre- and postmenopausal women. *Journal of Bone and Mineral Research*.

[12] Mödder UI, Hoey KA, Amin S (2011). Relation of age, gender, and bone mass to circulating sclerostin levels in women and men. *Journal of Bone and Mineral Research*.

[13] Boström P, Wu J, Jedrychowski MP (2012). A PGC1-α-dependent myokine that drives brown-fat-like development of white fat and thermogenesis. *Nature*.

[14] Spiegelman BM (2013). Banting lecture 2012: regulation of adipogenesis: toward new therapeutics for metabolic disease. *Diabetes*.

[15] Novelle MG, Contreras C, Romero-Picó A, López M, Diéguez C (2013). Irisin, two years later. *International Journal of Endocrinology*.

[16] Moreno-Navarrete JM, Ortega F, Serrano M (2013). Irisin is expressed and produced by human muscle and adipose tissue in association with obesity and insulin resistance. *The Journal of Clinical Endocrinology & Metabolism*.

[17] Huh JY, Panagiotou G, Mougios V (2012). FNDC5 and irisin in humans: I. Predictors of circulating concentrations in serum and plasma and II. mRNA expression and circulating concentrations in response to weight loss and exercise. *Metabolism: Clinical and Experimental*.

[18] Crujeiras AB, Pardo M, Arturo RR (2014). Longitudinal variation of circulating irisin after an energy restriction-induced weight loss and following weight regain in obese men and women. *American Journal of Human Biology*.

[19] American Diabetes Association (2013). Standards of medical care in diabetes—2013. *Diabetes Care*.

[20] Schoenau E (2005). From mechanostat theory to development of the “functional muscle-bone-unit”. *Journal of Musculoskeletal Neuronal Interactions*.

[21] Rahman S, Lu Y, Czernik PJ, Rosen CJ, Enerback S, Lecka-Czernik B (2013). Inducible brown adipose tissue, or beige fat, is anabolic for the skeleton. *Endocrinology*.

[22] Amrein K, Amrein S, Drexler C (2012). Sclerostin and its association with physical activity, age, gender, body composition, and bone mineral content in healthy adults. *Journal of Clinical Endocrinology and Metabolism*.

[23] Wells JCK (2007). Sexual dimorphism of body composition. *Best Practice and Research: Clinical Endocrinology and Metabolism*.

[24] Davis SR, Walker KZ, Strauss BJ (2000). Effects of estradiol with and without testosterone on body composition and relationships with lipids in postmenopausal women. *Menopause*.

[25] Mirza FS, Padhi ID, Raisz LG, Lorenzo JA (2010). Serum sclerostin levels negatively correlate with parathyroid hormone levels and free estrogen index in postmenopausal women. *Journal of Clinical Endocrinology and Metabolism*.

[26] Mödder UI, Clowes JA, Hoey K (2011). Regulation of circulating sclerostin levels by sex steroids in women and in men. *Journal of Bone and Mineral Research*.

[27] Al-Daghri NM, Alkharfy KM, Rahman S (2014). Irisin as a predictor of glucose metabolism in children: sexually dimorphic effects. *European Journal of Clinical Investigation*.

[28] Drake MT, Srinivasan B, Mödder UI (2010). Effects of parathyroid hormone treatment on circulating sclerostin levels in postmenopausal women. *Journal of Clinical Endocrinology and Metabolism*.

